# Biosynthesis and the Roles of Plant Sterols in Development and Stress Responses

**DOI:** 10.3390/ijms23042332

**Published:** 2022-02-20

**Authors:** Yinglin Du, Xizhe Fu, Yiyang Chu, Peiwen Wu, Ye Liu, Lili Ma, Huiqin Tian, Benzhong Zhu

**Affiliations:** 1 The College of Food Science and Nutritional Engineering, China Agricultural University, Beijing 100083, China; dyl0806@cau.edu.cn (Y.D.); cyy2293@cau.edu.cn (Y.C.); wpw1026@126.com (P.W.); liuliuye7@163.com (Y.L.); 17736231819@163.com (L.M.); chenfl@cau.edu.cn (H.T.); 2 The College of Biosystems Engineering and Food Science, Zhejiang University, Hangzhou 310012, China; xizhefu@zju.edu.cn

**Keywords:** plant sterols, biosynthesis, growth and development, regulation, stress responses

## Abstract

Plant sterols are important components of the cell membrane and lipid rafts, which play a crucial role in various physiological and biochemical processes during development and stress resistance in plants. In recent years, many studies in higher plants have been reported in the biosynthesis pathway of plant sterols, whereas the knowledge about the regulation and accumulation of sterols is not well understood. In this review, we summarize and discuss the recent findings in the field of plant sterols, including their biosynthesis, regulation, functions, as well as the mechanism involved in abiotic stress responses. These studies provide better knowledge on the synthesis and regulation of sterols, and the review also aimed to provide new insights for the global role of sterols, which is liable to benefit future research on the development and abiotic stress tolerance in plant.

## 1. Introduction

**Figure 1:**
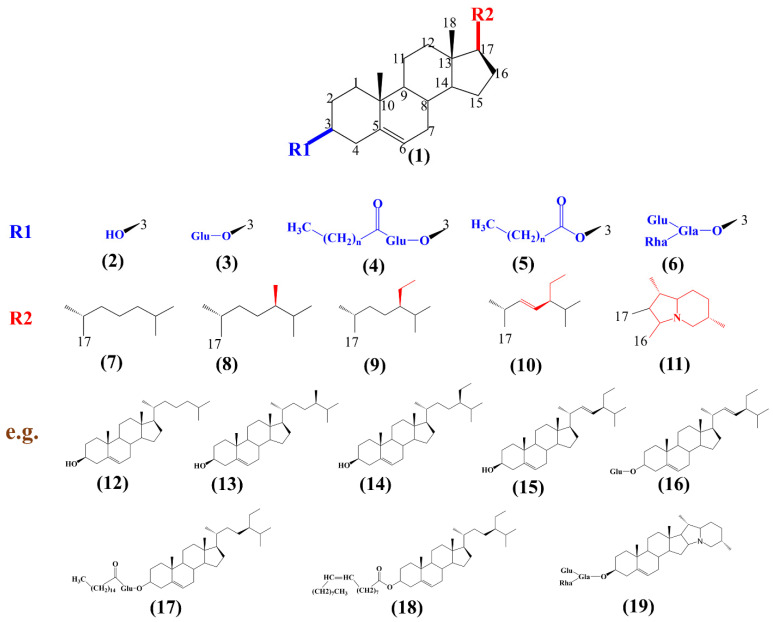
The chemical structure of free sterols and conjugated sterol. R1 at C3 and R2 at C17 determine the diversity of sterols. (**1**) Tetracyclic skeleton of sterol; R1 determines the type of compound including, (**2**) free sterol, (**3**) steryl glucosides, (**4**) acylated steryl glucosides, (**5**) steryl esters, (**6**) steroidal glycoalkaloids. R2 determines the diversity of compounds. For example, the combination of (**2**) and (**7**) makes cholesterol (**12**), the combination of (**2**) and (**8**) makes campesterol (**13**), the combination of (**2**) and (**9**) makes β-sitosterol (**14**), the combination of (**2**) and (**10**) makes stigmasterol (**15**), the combination of (**3**) and (**10**) makes stigmasteryl glycose (**16**), the combination of (**4**) and (**9**) makes sitosteryl palmitoyl glucoside (**17**), the combination of (**5**) and (**9**) makes stigmasteryloleate (**18**), while the combination of (**6**) and (**11**) makes α-solanine (**19**). The differences in the R1 group and R2 group are shown in blue and red, respectively.

Sterols are a kind of isopentenyl pyrophosphate-derived molecule, and exist naturally with important physiological activities for all eukaryotes [1]. Depending on their different sources, sterols can be divided into animal sterols, plant sterols, and fungal sterols [2]. At present, more than 250 kinds of sterols and sterol conjugates have been identified in plants, including free sterols, sterol esters, sterol glucosides, and acylated sterol glucosides (Figure 1), and the multiple forms of sterols determine their various functions in plant life [3,4].

The physiological and biochemical functions of kinds of plant sterols have been clarified in plants [5]. Firstly, as integral components of the membrane, plant sterols contribute to maintaining the integrity, fluidity, and permeability of the membrane lipid bilayer, thereby increasing the stress resistance of plants [6,7]. In addition, plant sterols participate in the formation of lipid rafts, a special structure, which is helpful to the establishment of cellular polarity, signaling, and plant–pathogen interactions [8,9]. Furthermore, plant sterols and their derivative brassinosteroid (BR) also serve important physiological functions in plant growth and development such as seed germination, plant phenotype, senescence, flowering time, and seed yield [10,11].

In view of the fact that plant sterols play an essential role in many crucial aspects and their future promising applications, here we provide an overview of the frontier advances in plant sterol biosynthesis, metabolism and stress response. Furthermore, we explore the potential mechanisms of plants regulating C24-alkyl sterols content in response to environmental stress and aims to provide new insights into understanding the role of sterol in cellular metabolism.

## 2. Key Enzymes in the Biosynthesis of Sterols in Plant

**Figure 2:**
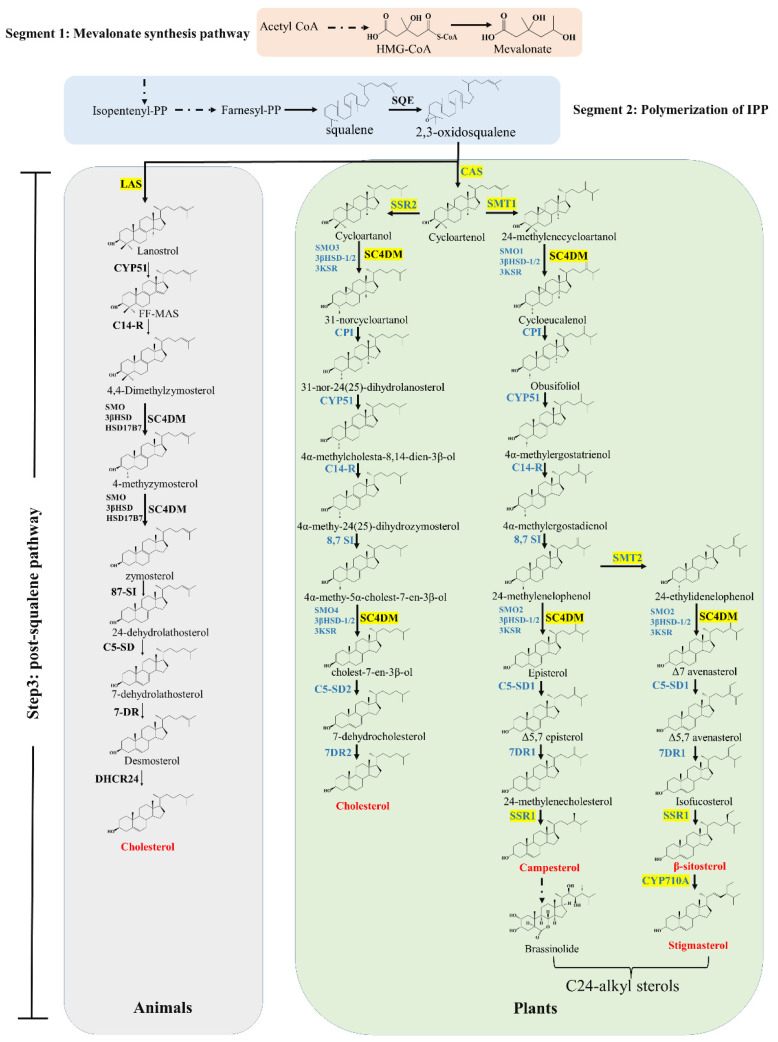
Biosynthesis pathways of plant sterols. Enzymes of the plant sterols synthesis pathway are marked in blue, the key enzymes that regulate C24-alkyl sterol synthesis are depicted in yellow, major sterols in plants are marked in red, and dotted lines represent multiple reaction steps. Species names are abbreviated as follows: FDFT1, farnesyl-diphosphate farnesyltransferase; SQE, squalene monooxygenase; LAS, lanosterol synthase; CYP51; sterol C-14 demethylase; C14-R, sterol C-14 reductase; SC4DM, sterol C-4 demethylation complex enzymes (include SMO, 4-methyl sterol oxidase; 3β-HSD, 3β-hydroxysteroid dehydrogenase; 3KSR, 3-keto sterol reductase) ; 8,7-SI, sterol 8,7 isomerase; C5-SD, sterol C-5 desaturase; 7-DR, 7-dehydrocholesterol; DHCR24, Δ(24)-sterol reductase; CAS, cycloartenol synthase; SMT, sterol C24-methyl transferase; CPI, cyclopropyl sterol isomerase; SSR, sterol side-chain reductase; CYP710A, sterol C-22 desaturase.

To date, a growing number of studies have been performed in the field of sterol biosynthesis [12,13]. This biosynthesis pathway can be divided into three major segments: (i) the mevalonate synthesis pathway, (ii) polymerization of isopentenyl pyrophosphate (IPP), and (iii) the post-squalene pathway (Figure 2). The first two segments are conserved in all eukaryotes, whereas profound differences exist in the post-squalene pathway in animals, plants, and fungi. Thus, in this section, we aimed to summarize key enzymes in the post-squalene pathway of the plant sterols biosynthesis, which is crucial for plant responses to abiotic stress, mainly through maintaining the optimal balance between cholesterol and C24-alkyl sterols in plants, as well as changes in the ratio between sterols and sphingolipids [14].

### 2.1. Cycloartenol Synthase 

Cycloartenol synthase (CAS, EC: 5.4.99.8) is a unique oxidosqualene cyclase in plants, which can catalyze the cyclization of 2,3-oxidosqualene to cycloartenol, a pentacyclic triterpenoid, serving as a common precursor for cholesterol and C24-alkyl sterols in plants. In this process, CAS can transfer the cation at position C-20 of oxidized squalene to position C-9 to form a cyclopropane ring between C-9 and C-19. Recently, CAS was known to significantly influence the sterols content, and subsequently cause phenotypic changes, via the regulation of *CAS* gene expression. For example, *CAS*-silenced tomatoes have shown a drastic reduction in cycloartenol, cholesterol, and C24-alkyl sterols levels [15]. Interestingly, the down-regulation of *NbCAS1* expression significantly altered the transcriptional expression of other genes in the plant sterol synthesis pathway, such as the enhanced expression of *NbHMGR1* and the weakened expression of *NbSSR2* and *NbSMT1* in *Nicotiana benthamiana* *(N. benthamiana*) leaves [16]. In addition, the *Arabidopsis thaliana* (*A. thaliana*) *cas1-1* mutant with a low *CAS1* expression, 2,3-oxidosqualene accumulation was found in all analyzed organs, which resulted in a slightly albino phenotype of inflorescence shoots that affects chlorophyll differentiation and pigment accumulation [17]. Hence, these studies demonstrated that CAS has a substantial impact on plant sterol biosynthesis and other physiological and biochemical processes including pigment synthesis.

### 2.2. Lanosterol Synthase

Lanosterol synthase (LAS, EC: 5.4.99.7) is also an oxidosqualene cyclase responsible for the cyclisation of 2,3-oxosqualene to lanosterol mainly in animals and fungi, as a key precursor for cholesterol [18,19,20]. It had long been believed that plants synthesize sterols only through cycloartenol rather than lanosterol [21]. However, recent research updated this review with new evidence. Ohyama et al. made use of isotope tracing technology to prove that plant sterols can be synthesized via both lanosterol and cycloartenol pathway in *A. thaliana* plant, whose contribution to the production of sterols are 99% and 1%, respectively [12]. In potato leaves with the over-expression of *LAS*, the glycoalkaloid content increased significantly, while the content of campesterol and sitosterol rapid decreased [22]. However, Elisabet et al. found that the silencing of *LAS* in *N. benthamiana* with VIGS showed no significant changes in sterol content, suggesting that sterol biosynthesis in *N. benthamiana* was strictly dependent on the CAS pathway [23]. Moreover, the same result was observed in *LAS*-silenced tomato plants [15]. Thus, it is entirely reasonable to consider that LAS exists ubiquitously in eukaryotes, while illustrating various contributions to sterol synthesis in different plants.

### 2.3. Sterol Side Chain Reductase

In plants, sterol side chain reductase (SSR, EC: 1.3.1.72) is well-known as being divided into two categories, Δ24-sterol reductase (SSR1), and sterol side chain reductase 2 (SSR2) [24]. Previous studies have reported that SSR1 catalyzed the last step in the synthesis of both campesterol and β-sitosterol, while SSR2 is only responsible for the conversion of cycloartenol to cycloartenol in the cholesterol pathway [25,26]. SSR1 is also named as DFW1, which is largely reported to affect the vegetative and reproductive growth in various kinds of plant [27]. For instance, the overexpression of *PcDWF1* in tobacco promoted the growth of stems and roots, as well as delayed flowering time [28]. In addition to acting as an important regulator in plant growth and development, SSR1 also affects the level of auxin in plants. In *SSR1* knock-out *A. thaliana* mutant, the auxin level is significantly reduced, resulting in the dramatic inhibition of primary root growth [26]. Notably, the *dwf1* plants with SSR1 loss-of-function often exhibited a dwarf phenotype, which has been confirmed as a brassinosteroid (BR)-deficient effect [29]. Different from the role of SSR1 on C24-alkyl sterol biosynthesis, SSR2 mainly participates in the synthesis of cholesterol in the plant, responsible for catalyzing the conversion of cycloartenol to cycloartanol [30]. It has been proven that SSR2 regulation in the plant causes the change in cholesterol content, and subsequently influenced the accumulation of steroidal glycoalkaloids (SGAs), which is known as an important anti-insect substance. In *SSR2*-silenced potato plants, the levels of cholesterol and SGAs were greatly lower than that of wild type, without a distinct effect on plant growth or yields [30,31]. Hence, SSR1 and SSR2 are both important sterol side chain reductases, required for the biosynthesis of cholesterol and C24-alkyl sterols, respectively.

### 2.4. Sterol Methyltransferase

As we know, the presence of alkyl groups at C-24 sterol is the key difference point between plant sterols and animal sterols [14]. In plants, the alkylation of the side chain is catalyzed by sterol methyltransferase (SMT) [32]. SMT is essential for the formation diversity of plant sterols, responsible for the orientation of post-squalene metabolic fluxes towards the biosynthesis of cholesterol, campesterol, and sitosterol [5,33]. There are two types of SMT in plants, namely SMT1 (EC: 2.1.1.41) and SMT2 (EC: 2.1.1.143), involved in primary methylation and secondary methylation, respectively [34]. SMT1 catalyzes the methylation of cycloartenol to 24-methylene cycloartenol, which is the first step in introducing methyl groups in the 24-desmethyl sterols. In *SMT1*-silenced *Withania somnifera* (*W. somnifera*) plants, the down-regulation of *SMT1* expression resulted in the reduction in campesterol, sitosterol, and stigmasterol levels but also an increase in cholesterol content [35]. In addition, it has been found that the enhancement of SMT1 activity in overexpressing *SMT1* tobacco promotes an increase in endogenous 3-hydroxy-3-methylglutaryl coenzyme A reductase (HMG-CoA) activity, which involves IPP polymerization in the sterol biosynthesis pathway [36]. SMT2 catalyzed the conversion of 24-methylenelophenol to 24-ethylidenelophenol. It is noteworthy that 24-methylenelophenol is the branch point of C24-methyl sterols and C24-ethyl sterols synthesis [37]. Anjanasree et al. observed that in *smt2* mutant, the contents of downstream branch products of sitosterol and campesterol were decreased, while the 24-methylenelophenol level was increased relative to that of the wild type [38]. In addition to affecting the production of C24-alkyl sterols, SMT2 is also required for the ploidy consistency of the sexual reproduction system in *A. thaliana*. In *smt2 A. thaliana* mutant plants, weak cytokinetic defects appear in the flowers, accompanied by the erroneous generation of tetraploid meiocytes [39]. Furthermore, upstream promoter sequence analysis of *SMT2* in soybean revealed several important binding sites for temperature-related transcription factors, indicating that SMT2 may help plants cope with wider temperature fluctuations and maintain membrane-related metabolic processes, thereby regulating plant growth and development [40].

### 2.5. Sterol C-4 Demethylation Complex Enzymes (SC4DM)

C-4 demethylation of sterol intermediates (SBIs) is a critical step in sterol biosynthesis, which is catalyzed by sterol C-4 demethylation complex enzymes (SC4DM) that are highly conserved from animals to plants [41,42]. Undoubtedly, the conversion of C4-methyl sterols into functional sterols only occurs after the removal of the two methyl groups at the C-4 position [42,43]. Previous studies have shown that demethylation reaction is thought to proceed by means of a three-steps mechanism: (i) oxidation of the methyl group into a carboxyl group at the C-4 position by 4-methyl sterol oxidase (SMO, EC: 1.14.18.10), (ii) oxidation of the hydroxyl group into a ketone group at the C-3 position and decarboxylation at C-4 position by 3β-hydroxysteroid dehydrogenase (3β-HSD, EC: 1.1.1.170), (iii) reduction of the ketone group into a hydroxyl group at the C-3 position by 3-keto sterol reductase (3KSR, EC: 1.1.1.270) [44,45].

In animals, a single SMO enzyme is involved in two consecutive demethylation steps [42]. However, in plants, it was suggested that SMO1 and SMO2 remove the first and second C-4 methyl group of sterols in C24-alkyl sterol biosynthesis [42]. In addition, SMO3 and SMO4 are involved in the first and second C-4 demethylation of cholesterol precursors in plants [15]. Currently, 3β-HSD in plants has been verified as being a common enzyme in the biosynthesis of both C24-alkyl and cholesterol in plants. However, due to the lack of protein homology with 3KSR in other species, such as humans, mice, and yeast, obviously it cannot easily be screened by traditional methods including evolutionary proteomics, phylogenomics, transcriptomics, and proteomics co-expression analysis and still unable to be clearly annotated in KEGG. Thus, the composition and efficacy of SC4DM need to be further studied.

### 2.6. C22-Sterol Desaturase

**Figure 3:**
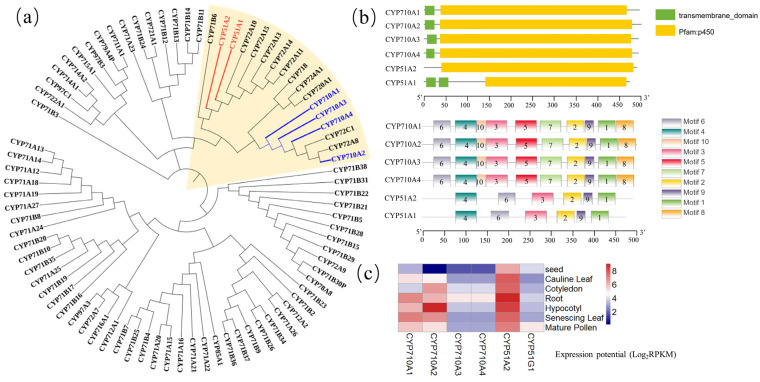
(**a**) Phylogeny of the *CYP* gene superfamily in *A. thaliana*. The unrooted phylogenetic tree of 81 *CYP* genes in *A. thaliana* was constructed by MEGA7.0 with the neighbor-joining (NJ) method. In the phylogenetic tree, *CYP710* is marked in blue, *CYP51* is marked in red, *CYP710* and *CYP51* cluster into one clade is marked in yellow. (**b**) The domain and motif of *CYP710* and *CYP51* were analyzed by NCBI and MEME, respectively. (**c**) Expression profile of *CYP710* and *CYP51* in different tissues of *A. thaliana*.

C22-sterol desaturase (CYP710, EC: 1.14.19.41) has been widely characterized to catalyze the synthesis of stigmasterol from *β*-sitosterol [46,47,48]. Overexpressing *CYP710A1* and *CYP710A11* in *A. thaliana* plants resulted in the content of stigmasterol increasing by more than six-fold [46]. New research indicated that *CYP710A* overexpression in *W. somnifera* not only significantly upregulated the level of stigmasterol, but also promoted accumulation of withanolides [49]. Notably, CYP710 belongs to the Cytochrome P450 superfamily, the largest enzyme family in plants which has been reported to participate in a variety of complex biological metabolic pathways and play crucial roles in plant development and stress tolerance [49,50,51]. Interestingly, CYP51, similarly to *CYP710*, belongs to the Cytochrome P450 protein family, and it catalyzes an essential early step in sterol metabolism, removing a methyl group from obtusifoliol and 31-nor-24(25)-dihydrolanosterol in plants [52]. Here, a phylogenetic tree of the Cytochrome P450 protein family (related to terpenoid synthesis) in *A. thaliana* was constructed to find that CYP51 and CYP710 are clustered together (Figure 3a). Furthermore, CYP710 and CYP51 are similar in conserved protein domains, motifs and gene expression patterns (Figure 3), suggesting that CYP710 might be evolved from CYP51, and might have the function of catalyzing demethylation of sterols and forming double bonds.

## 3. Roles of Sterols in Plant Growth and Development

Numerous previous reports indicated that plant sterols are involved in the regulation of plant growth and development [5,53]. In this section, an overview of sterols’ roles is mainly shown below with seed germination, plant architecture, and plant reproduction growth.

### 3.1. Seed Germination

Seed germination is an indispensable process, which is helpful for plants growing from a single seed into a plant, as well as affecting both crop yield and quality [54].

Previous studies have revealed that changes in sterol contents can significantly affect seed germination. For instance, the *hise1-3 psat1-2* double mutant *A. thaliana* plants showed an excessive accumulation of sterols, consequently resulting in delayed seed germination and a thick seed coat [10]. Due to the lack of sterols in *A. thaliana dwf5* and *dwf7* mutants plant, the seed germination is strictly inhibited, and the morphology of seed is notably altered [55]. Additionally, *HMGS*-overexpressing *A. thaliana* seedling were reported to accelerate seed germination, show much higher sterol content and enhanced *Botrytis cinerea* resistance when compared with wide type Arabidopsis plants. Additionally, overexpression of *HMGS* significantly upregulated the expression of sterol synthesis-related genes including *HMGR*, *SMT2*, *DWF1*, and *CYP710A1* in *A. thaliana* [56]. Furthermore, overexpressing *3βHSD1* (a gene member of SC4DM) in *A. thaliana* led to the reduction in dormancy compared with that of the wild type [57]. In summary, these results suggest that plant sterols play an essential role as a promotor in seed germination under normal conditions.

### 3.2. Plant Architecture

The study of plant architecture is one of the most popular fields in plant developmental biology [58]. The architecture of higher plants shows great diversity, which is determined by the relative arrangement of leaves, flowers, branches, and roots [59,60]. At present, the research on the role of sterols in model plants such as Arabidopsis and tomato has greatly enhanced our understanding about the formation of plant architecture.

It was found that plant sterols such as C24-alkyl sterols, cycloartenol, and 4-carboxy-4-methyl-24-methylenecycloartanol (CMMC) are absolutely necessary to plant architecture. It is clear that sterols can regulate the architecture of plants by affecting the synthesis and transport of auxin. For example, the *A. thaliana* double mutant *smt2 smt3* exhibited a severe dwarfism phenotype throughout its life cycle, owing to significant inhibition of the directional auxin transport through the down-regulation of sitosterol and stigmasterol [37]. Similarly, CMMC, an intermediate in C4-demethylation of sterols, can also impacts phenotype as an inhibitor of auxin transport. Samba et al. observed that increased CMMC content in *A. thaliana erg28* mutant resulted in the inhibition of auxin transportation and led to abnormal phenotypes including dwarf plant, leaf fusion, and reduced root growth [44]. In addition, the short root phenotype of *cpi1-1* mutant *A. thaliana* was closely related to a markedly enhanced auxin response in the root tip, due to the up-regulated expression of auxin-synthesis-related genes *tryptophan aminotransferase of arabidopsis1* (*TAA1*) induced by cycloartenol accumulation and stigmasterol reduction [61]. Besides auxin, the cellulose content was also found to be regulated by sterols, which directly determines plant architecture. The reduction in cellulose content was associated with the hyper-cracking fruit phenotype of *Lycopersicon* *esculentum (L. esculentum*) mutant *3βhsd1*, which was influenced by the decreased content of plant sterols [62]. Additionally, *A. thaliana* plants treated with the sterol biosynthesis inhibitors 15-azasterol and fenpropimorph showed abnormal embryonic development and reduced cellulose levels [63].

Plant sterols also act as the precursors of brassinolide to affect plant architecture. The destruction of enzymes downstream of *SMT2* in the sterol biosynthesis pathway usually lead to campesterol depletion, consequently resulting in a severe dwarf phenotype, a typical phenotype of BR-deficient mutants [55]. This typical dwarf phenotype has been observed in many *smt2*-mutant plant species including cherry tomato [64], Arabidopsis [65], rice [66], and pea [67]. Interestingly, the dwarf phenotype can be restored by exogenous BR treatment in *Pisum sativum smt2* mutant, but not in *Cynodon dactylon* *smt1* mutant, for the latter dwarf phenotype is caused by the accumulation of spermine [68].

### 3.3. Plant Reproduction Growth

Plant reproduction growth comprises multiple processes such as flowering time, stigma development, pollen formation, and seed generation [69]. To date, many studies have proved that sterols play an irreplaceable role in these reproductive growth processes. For instance, in *A. thaliana ssr1* mutant, the production of sitosterol, stigmasterol, as well as campesterol was severely inhibited, which resulted in stigmatic developmental malformations and partial sterility [42]. In addition, the *A. thaliana smt1* mutant was reported to have a complex phenotype consisting of very low fertility, an altered flower morphology with serrated edges of petals and sepals, and the proliferation of secondary stems [5].

In addition to plant fertility, the regulation of sterol synthesis-related genes expression will also alter seed size and yield. A report by Suo et al. highlighted that overexpression of *GhSMT2-1* in *Gossypium hirsutum* significantly increased the size and weight of cotton seeds as well as the level of C-24 ethyl sterols [70]. In *A. thaliana*, *HMGS* overexpression improved seed yield and pod size, and also up-regulated sterol content [71]. Similarly, *HMGS*-overexpressing *Nicotiana tabacum* plants showed greater seed yield compared with that of the wild type [71]. Moreover, in *Oryza sativa* (*O. sativa*), knock out of *OsCYP51G1* resulted in reduced seed yield, delayed flowering, as well as abnormal pollen [72].

Notably, sterols can also participate in the formation of lipid rafts, sterol-rich PM microdomains, which play an essential role in the growth of cell polarity in pollen pores and tips of germinating pollen tubes [73,74]. Peng et al. demonstrated that the polarization of the sterol-rich PM microdomains to the top of the pollen tube was conducive to the formation of the pollen tube, which requires the participation of NADPH oxidase [75]. In addition, sterol chelator methyl-β-cyclodextrin (MβCD)-treated rice mature pollen resulted in the content of total sterol decreased by 50%, and destroyed the microdomain structure in the total pollen membrane, as well as significantly inhibiting the growth rate of pollen tubes [73]. Similarly, a reduced pollen growth rate was also observed in MβCD-treated tobacco pollen [76].

In summary, these studies demonstrate the indispensable roles of plant sterols in plant reproductive growth processes, especially in seed generation, which is helpful for substantially increasing crop yields.

## 4. Sterols and Abiotic Stress Responses

The plant cell membrane is an important place to perceive stress signals. As essential components of the cell membrane, plant sterols are regarded as a requirement for plants in response to abiotic stress [6]. Previous studies have shown that stress can lead to changes in plant sterols content [7,77]. It is worth noting that plant sterols can also improve plant stress resistance mainly through exogenous sterol treatment or changes in endogenous sterol content. Plant sterols, especially C24-alkyl sterols, which have been reported to improve the resistance of plants through reinforcing the cohesion of cell membranes to change the permeability of membranes and affecting proton efflux [14,78,79]. In this section, we systematically summarize the role of sterols in plants’ responses to a variety of abiotic stress including temperature stress, salinity stress, UV stress, and drought stress (Table 1).

### 4.1. Temperature Stress

In plants, extreme temperature directly influences physiological activities such as photosynthesis, respiration, and transpiration, etc. [110,111]. It is well-known that sterol contents and the ratio of free sterols and sterol conjugates are markedly affected by extreme temperature in different plants. For example, Kazan et al. observed that the cold-induced expression of *TaCYP710A1* was higher in roots than that in leaves, and significantly increased the stigmasterol level in *Triticum aestivum* (*T. aestivum*) roots [80]. Under high-temperature conditions at 35 °C, thermal-tolerant *Lactuca sativa* (*L. sativa*) buds accumulated higher concentrations of campesterol, stigmasterol, and amino acids compared with those at 21 °C. Meanwhile, compared with thermal-tolerant *L. sativa* buds, thermal-sensitive *L. sativa* buds produced greater amounts of sterols during germination at 21 °C [81]. In contrast, high-temperature treatment significantly reduced the content of stigmasterol in *Helianthus annuus* during seed germination [82]. Additionally, in *Phaeodactylum tricornutum*, the total sterols content was significantly reduced at 23 °C when compared with that at 13 °C [83]. These studies indicate that sterols employ different metabolic styles in response to temperature stress during germination in different plants. Interestingly, the accumulation of phytosterols in *Corchorus depressus* was greater in winter than that in summer, which might be caused by the synergistic effect of temperature and other environmental factors such as drought [112]. Additionally, extreme temperature often affects the balance between free sterol and sterol conjugates. It was observed that low-temperature treatment induced the production of acylated sterol glycoside (ASG) and altered the ratio of free sterol and ASG in *Avena sativa* [84].

Importantly, the modification of sterol contents distinctly influences the cold and heat tolerance in plants. On the one hand, changes in endogenous sterol contents affect plant tolerance to different unsuitable temperatures. Muthappa et al. reported that under a high temperature, the promotion of stigmasterol content in *AtCYP710A1* overexpressing *A. thaliana* could significantly improve the heat tolerance and reduce the mortality of plant [85]. In addition, Valitova et al. observed that the reduction in sterol content by MβCD chelator treatment aggravated the cold stress injury of wheat roots, proving that sterols would enhance cold tolerance as well as the stability of the membrane in *T. aestivum* leaves [86]. On the other hand, previous studies have found that exogenous sterol treatments including the method of soaking seeds and foliar spraying could improve plant tolerance to cold and heat stress. For instance, tomato seeds soaked with 10 μM sitosterol showed distinctly promoted tolerance of tomato plants to both high and low temperature stress [87]. Similarly, the treatment of *Agrostis stolonifera* leaves with 400 μM sitosterol resulted in the inhibition of leaf senescence, the enhancement of membrane stability as well as the activation of plant antioxidant responses under heat stress, and ultimately enhanced plant heat tolerance [88].

Clearly, both elevated endogenous sterol content and exogenous sterol treatment increased plant tolerance to extreme temperatures, mainly through regulating the fluidity and permeability of the membrane. Below the phase transition temperature of the membrane, free plant sterols increase the fluidity of membrane lipids, while above the phase transition temperature, free plant sterols improve the ordering and cohesion of lipid bilayers as well as membrane permeability through the interaction with membrane lipids [113,114]. Consequently, this regulatory effect of sterols on the membrane ensures the adaptation of plant membrane systems to different temperatures.

### 4.2. Salinity Sress

Soil salinization is a major environmental problem affecting more than 800 million hectares of land around the world, which is equivalent to more than 6% of the world’s total land surface area [115]. Most crops have poor salt tolerance, especially major crops, such as wheat [116] and rice [117], showing significant yield reduction and other stress injuries under high salinity stress.

In addition to temperature stress, sterols are also involved in response to salt stress. Surjus et al. observed that the total sterol content has decreased by more than 50% in *Glycine max* under the treatment of 25 mM NaCl for 8 days [89]. Conversely, the content of total sterols significantly increased with high-salinity treatment in both salt-tolerant *Zea mays* and halophyte *Kosteletzkya virginica* [90,92]. Additionally, it has been reported that a reduction in sitosterol and an increase in stigmasterol appear in the plasma membrane (PM) of salt-treated *Brassica oleracea* (*B. oleracea*) roots [91]. Under all selected salinity levels (0, 170, 340 mmol/L), the major lipid remained stable with increasing salinity, but the increase in the relative percentage of campesterol and decrease in the relative percentage of sitosterol in response to elevated NaCl concentration in *Spartina patens* were also observed [93].

Notably, besides the change in sterol content when responding to high salinity stress, sterols can also enhance salt tolerance in plants [118]. For example, the up-regulation of campesterol and cholesterol content was significantly associated with improved salt tolerance in wheat [94]. Moreover, Kerkeb et al. demonstrated that an increase in the ratio of sterols and phospholipids under salt stress improved salt tolerance, with the exaltation of membrane rigidity in tomato callus [95]. In *AaSMO1*-overexpressing transgenic tobacco, it has been reported that increase in total sterol content induces the sensitivity of plants to dehydration stress [96]. Furthermore, it can be observed that a change in sterol content, especially the up-regulation of the stigmasterol level, resulted in the enhancement of the membrane adaption to salt stress in three different Brassicaceae species including *B. oleracea*, *Brassica napus*, and *Cakile maritima* [97].

In previous research, exogenous sterol treatment was shown to also improve plant salt tolerance. Hashem et al. proved that treatment of flax seeds with 200 ppm stigmasterol could increase the content of linolenic acid and oleic acid to overcome drastic effects such as an imbalance of endogenous hormones (IAA, GA_3_, and ABA) under salinity stress [98]. Moreover, treatment of *Capsicum annuum* leaves with 150 ppm sitosterol significantly offset the salinity stress damage such as electrolyte loss and inhibited growth, subsequently improving membrane stability and antioxidant enzyme activity [99].

### 4.3. UV Stress

According to different wavelengths, ultraviolet light can be divided into UV-A (315–400 nm), UV-B (280–315 nm), and UV-C (100–280 nm). Among them, UV-B is the most extensively studied form of ultraviolet light. It is clear that UV-B can induce a variety of damage effects such as chromosomal aberrations [119], cell disassembly, damage photosynthesis [120], reduced biomass, and teratogenic mutagenesis in plant morphology [121]. Previous studies have shown that plants respond to UV stress mainly through a complex series of biochemical reactions, including accumulation of UV-absorbing secondary metabolites such as inositol and flavonoids, to increase UV absorption and neutralize the reactive oxygen species produced by UV [122].

Recent studies have shown that sterols are involved in responding to UV damage. For example, the contents of sitosterol, stigmasterol, as well as lupeol sterol were up-regulated with low-intensity UV-B treatment (8.25 lW·cm^−2^, 16 h) in *Vitis vinifera* leaves. [100]. Although the contents of triterpenoid were also increased by high UV-B (33 lW·cm^−2^, 4 h) treatment, the increase was not as pronounced as low radiation conditions [100]. This indicates that low-dose UV-B treatment promotes a greater production of triterpene sterols than that of high-dose UV-B treatment. However, some other compounds with antioxidant properties such as diterpenes and tocopherols were significantly increased after high UV-B irradiation, while not observed in the low UV-B group. This accumulation of antioxidant alkenes as well as ABA in the high UV-B treatment group stimulates the initiation of the plant defense system, which have been largely found in the study of grape resistance to UV damage [123,124]. By contrast, different doses of UV-B irradiation treatment for *Olea europaea* increased the accumulation of ursolic acid and lupeol, while did not significantly influence sterol content [101]. Furthermore, after treatment with UV-B (3.6 kJ·m^−2^·day^−1^) in *W. somnifera*, it was found that triterpenoid sterols showed a significant decrease in treated leaves at all sampling ages, while a completely opposite trend was observed in roots [102]. Hence, these results show that the regulation of sterol synthesis in response to UV-B stress is significantly distinct in different tissues and plants, indicating the complex mechanism of plant sterols in response to UV-B stress.

There are a few studies on the effect of exogenous sterol treatment on plant UV-tolerance. Raheel et al. demonstrated that exogenous application of β-sitosterol has a positive effect on the growth of *O. sativa* plants and the tolerance to UV-B stress. The length of roots and stems as well as biomass sharply decreased by more than 50% in the control group compared with that of exogenous sitosterol application group under UV-B radiation stress, which exhibited no significant changes [103].

### 4.4. Drought Stress

Drought stress is one of the main abiotic stresses that directly affect plant growth and limit the yield of plants [125]. It has been reported that sterols can also generate a response to drought stress. For example, it can be observed that the main sterols, including stigmasterol, campesterol, and β-sitosterol, gradually increased in rice during the treatment period of drought stress [104]. In addition, an increase in plant sterol content, especially β-sitosterol, was observed in *C. pepo* under drought stress, while the oil production of pumpkin seeds was inhibited [105]. Sujith Kumar et al. also found that drought treatment distinctly increased the production of both free sterols and sterol ester in *T. aestivum*. Under drought conditions, the expression levels of sterol synthesis and metabolism-related genes, such as *HMGH* and *PSAT,* were up-regulated with the extent directly proportional to the severity of drought stress [106]. Furthermore, sterols were significantly accumulated in the leaves of tea seedlings of different maturity under water deprivation treatment [107].

Of course, changes in sterol content will also affect the drought tolerance of plants. Chen et al. reported that the down-regulated expression of *OsSMT1* and *CdSMT1-1* resulted in the accumulation of cholesterol and a reduction in C24-akyl sterol content, as well as increase in polyamine content and the enhancement of drought tolerance in transgenic *O. sativa* and *Cynodon dactylon* [68]. Consistent with other abiotic stress, exogenous sterol treatment also improves the drought tolerance of plants. For example, the drought tolerance and total antioxidant capacity were simultaneously and significantly improved in *Trifolium repens* with 120 μM sitosterol treatment [108]. Additionally, 100 μM sitosterol treatment on *T. aestivum* could reduce the damage caused under drought conditions, such as the loss of yield and inhibition of growth when compared with the control group [109].

## 5. Molecular Regulation of Plant Sterols

In the above section, we have introduced the important role of plant sterols in plant growth and development and stress resistance in detail [7]. In this section, we generalize the current research on the molecular regulation of plant sterol synthesis, which mainly focuses on two aspects, including transcriptional regulation and post-translational regulation.

### 5.1. Transcriptional Regulation

Previous studies have shown that transcription factors including WRKY TFs, MYC TFs, and ERF TFs regulate the synthesis of sterols in plants (see Table 2 for details).

WRKY transcription factors, one of the largest families of transcriptional regulators in plants, are unique zinc finger-type transcriptional regulators in plants. WRKY factors were named for containing a highly conserved seven amino acid sequence, WRKYGQK, at their N-terminus [132]. It has been found that WRKY TFs can specifically bind to the (T)(T)TGAC(C/T) sequence (also known as W-box) [133], and also function as salicylic acid response factors involved in biotic and abiotic stresses [134]. WRKY1 belongs to WRKY TFs and participates in regulating the levels of secondary metabolites such as sterols and glycoalkaloids in plants. For example, *WsWRKY1* silencing resulted in the hindrance of plant growth and decreased plant sterol content, while overexpression of *WsWRKY1* promoted the synthesis of plant sterols in *W. somnifera*, tobacco, and tomato [127]. Furthermore, with the overexpression of *PqWRKY1* in *A. thaliana*, the expression of plant sterols synthesis-related genes (such as *HMGR, FPS2, SQS1,* and *SQE2*) in transgenic line increased by one- to five-fold compared with that of the control line [128].

MYC transcription factor is an important member of the bHLH family, which consists of a basic domain binding to DNA at the N-terminus, and the bHLH domain containing two amphipathic α helices at the C-terminus [135,136]. In addition, MYC transcription factor is a principal component in the jasmonic acid regulatory network, which is involved in the regulation of many processes of plant development and stress resistance [137]. Similarly to WRKY TFs, MYC TFs, especially the MYC2 TFs, have also been associated with the synthesis of sterols in plants. For example, overexpression of *WsMYC2* in the leaves of *W. somnifera* significantly increased the expression levels of *WsCYP710A* and *WsCAS*, while the content of stigmasterol increased by 1.5-fold and 1.33-fold with jasmonic acid treatment on the third and sixth day, respectively. In addition, the *WsMYC2* knockout mutant also resulted in a significant reduction in the stigmasterol content [129]. Functional validation revealed that *TwMYC2a* and *TwMYC2b* were negative regulators of sterol synthesis in *Tripterygium wilfordii* (*T. wilfordii*). Silencing *TwMYC2* up-regulated the expression of *TwHMGR1* by 1.64-fold and increased triptolide content by 1.67-fold in roots [130]. Interestingly, MYC2 has opposing regulatory functions on sterol synthesis in *T. wilfordii* and *W. somnifera*, indicating that the role of MYC2 in plant sterol synthesis still needs further investigation.

Ethylene response factor (ERF) is a member of the AP2/ERF transcription factor superfamily in plants [138]. It is clear that the structure of ERF is characterized by an AP2 domain, three reverse parallel β-folds, and an almost parallel α helix [139,140]. Studies have shown that a kind of jasmonate-responsive ERF4 participates in regulating the expression of sterol synthesis-related genes from the upstream mevalonate pathway to cholesterol biosynthesis and downstream aglycone formation and glycosylation in tomato [131]. Additionally, ERF is a positive regulator in the process of alkaloid biosynthesis in tobacco. Ectopic overexpression of *NtERF32* in BY-2 cells increased the expression of *NtPMT1a* and promoted the accumulation of the total alkaloid content, whereas down-regulation of *NtERF32* expression significantly inhibited the level of nicotine and total alkaloid [141]. Similarly, ORC1 is a member of AP2/ERF factors, and overexpression of *ORC1* could promote the synthesis of alkaloids in *Nicotiana glauca* [142].

There are a few studies on transcription factors that regulate sterols synthesis in plants. Only three kinds of transcription factors have been found to be associated with sterols biosynthesis in plants, including WYRK TFs, MYC TFs, and ERF TFs. Therefore, others transcription factors which are responsible for plant sterols biosynthesis are required for numerous studies in the future.

### 5.2. Post-Translational Regulation

Research about the post-transcriptional regulation of sterols is mainly focused on hydroxymethylglutaryl-CoA reductase (HMGR), a rate-limiting enzyme in the plant sterols biosynthetic pathway [143]. In the past, the post-translational regulation of HMGR included phosphorylation, glycosylation, ubiquitin, and protein degradation [144,145,146]. Recently, a new negative regulator HIGH STEROL ESTER1 (HISE1) was found in *A. thaliana*, which can significantly reduce protein activity of *HMGR1* and *HMGR2*, thus inhibiting the overproduction of sterols. There was no obvious change in the expression levels of *HMGR1* and *HMGR2* at the mRNA level in the *hise1-2* mutant, while the specific activity of HMGR in the extract of leaf was about 23-fold higher than the wild type, which indicates that the regulation of HMGR by HISE1 occurred at the post-transcriptional level. Furthermore, the contents of cycloartenol and 24-methylenecycloartanol are up-regulated in the *hise1-2* mutant, which further proved that HISE1 is a negative regulator of sterol synthesis in plants [147].

In addition to regulating the sterol content via post-translational regulation of HISE1, plants can also alter the levels of sterols by converting sterols into sterol esters. It has been proved that excessive sterols are detoxified through conversion to sterol esters by phospholipid sterol acyltransferase 1 (PSAT1) in the ER microdomains, and subsequently separated in the SE bodies [148]. Therefore, the self-balance of sterols depends on the regulation of HISE1 and PSAT1, which is essential for maintaining sterols at normal levels in plants.

## 6. Conclusions and Future Trends

As a kind of crucial cellular membrane compound, sterols not only regulate the growth and development of plants, but are also involved in the plant response to abiotic stress. Recently, this research on plant sterols has progressed substantially. In this paper, recent findings in the field of plant sterols have been systematically summarized, including their biosynthesis, regulation, as well as functions. We review the key enzymes in the biosynthesis of sterols in plants including LAS, CAS, SMT, SSR, SC4DM, and CYP710, which aimed to make it possible to control the expression level of key enzyme genes in the synthesis pathway. It would be helpful for using modern molecular biology methods to cultivate new genetically modified varieties with high levels of plant sterols.

In addition, we summarized the role of sterols in seed germination, plants architecture, and plant reproduction growth. Through the analysis of previous studies, we found that the sterol-deficient mutants displayed a defective growth phenotype, which was attributed to the detrimental effects of cellulose synthesis [149]. Clearly, the level of cellulose is mainly positively regulated by sitosterol glycoside, which belongs to sterol glycoside, as a conjugated sterol in plants [150]. However, in the latest study, Takshak et al. found that sterol glycoside, notably sitosterol and stigmasterol glucosides, negatively regulated cellulose synthesis in *W. somnifera* [151]. These results suggest that the molecular regulation of sterols and conjugated sterol in plants is a complex system, and the regulatory network of plant sterol signaling molecules remains to be further studied.

**Figure 4:**
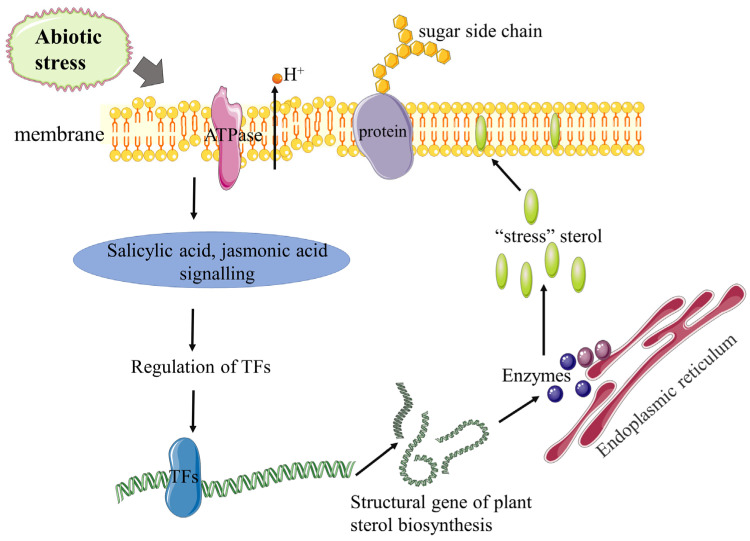
Role of plant sterols in response to abiotic stress. Under abiotic stress, the structure of the membrane is destroyed, which affects the permeability of the membrane and the activity of the membrane-bound protein, causing the loss of H^+^. Transcription factors are regulated by corresponding hormone signals to regulate the expression of structural genes of sterol biosynthesis to affect the content of “stress” sterols and ultimately maintain membrane stability.

Furthermore, there are a few studies on transcription factors related to sterol synthesis in plants only including WRKY TFs, MYC TFs, and ERF TFs. It is noteworthy that WRKY1 is a salicylic acid response factor, while MYC2 and ERF4 are both jasmonic acid response factors, which are all involved in regulating the synthesis of sterols in plants. Therefore, we propose that plants regulate the changes in sterols, especially C24-alkyl sterol content, in response to environmental stress, which may be implicated in hormone induction (Figure 4). At present, there are no related reports on post-transcriptional regulation and epigenetic regulation of plant sterols biosynthesis, and this regulation of sterols in plants requires further study. In conclusion, this review will provide more clues for the biosynthesis and regulation of plant sterols, the analysis of which might assist in revealing the metabolic activity of regulatory networks and further enhance the environmental stress tolerance of plants.

## Figures and Tables

**Table 1:** The role of sterols in plant responses to a variety of abiotic stress.

Plant	Related Treatment	Stress Factor	Effect	References
*Triticum aestivum*		4 °C for 1 and 12 h	Up-regulated the expression of *TaCYP710A1*; increased the content of stigmasterol	[80]
*Lactuca sativa*		35 or 21 °C for 40 h	Up-regulated the concentrations of sterols	[81]
*Helianthus annuus*		25 or 35 °C for 1–5 days	Reduced the level of stigmasterol	[82]
*Phaeodactylum tricornutum*		13 or 23 °C under white light	Reduced the level of the total sterols at 23 °C	[83]
*Avena sativa*		2 °C for 4 weeks	Increased acylated sterol glycoside (ASG) content; changes in the ratio of free sterol and ASG	[84]
*Arabidopsis thaliana*		7 ± 1 ℃ for 30 days	Increased the permeability of plant cell membrane; increased ratio of stigmasterol to sitosterol	[85]
*Arabidopsis thaliana*		45 ℃ for 3 and 6 h	Increased fatality rate	[85]
*Arabidopsis thaliana*	AtCYP710A1 gene-overexpression	45 ℃ for 3 and 6 h	Enhanced the heat tolerance and reduced the mortality	[85]
*Triticum aestivum*		4 ℃ for 1 and 12 h	Destructed membrane integrity; accumulated reactive oxygen species; increased total sterol content; increased the ratio of C24-methy sterol and C24-ethyl sterol	[86]
*Triticum aestivum*	5 mM MβCD for 12 h	4 ℃ for 1 and 12 h	Decreased sterol content; aggravated the cold stress injury	[86]
*Lycopersicon esculentum*	Seeds were soaked with 10 μM sitosterol for 10 h	10, 25, and 45 ± 3 °C for 14 days	Increased tolerance of tomato plants to both high and low temperature stress	[87]
*Agrostis stolonifera*	400 μM sitosterol, foliar spraying until droplets formed	35 °C for 28 days	Inhibited the leaf senescence under heat stress; enhanced plant heat tolerance.	[88]
*Glycine max*		25 mM NaCl for 8 days	Decreased the content of total sterols by 50%; increased the content of saturated fatty acids	[89]
*Kosteletzkya virginica*		85 mM NaCl for 17–20 days	Decreased the content of total sterol	[90]
*Brassica oleracea*		0, 40 and 80 mM NaCl for 15 days	Decreased the content of sitosterol; increased stigmasterol content	[91]
*Zea mays*		150 mM NaCl for 15 days	Decreased the content of total sterol	[92]
*Spartina patens*		0, 170, 340, and 510 mM NaCl for 10 weeks	Decreased sitosterol in response to elevated NaCl.	[93]
*Triticum aestivum*		150 mM NaCl for 21 days	Decreased the content of campesterol and cholesterol; improved salt tolerance	[94]
*Lycopersicon esculentum*		50 and 100 mM NaCl for 4 weeks	Increased the ratio of sterols and phospholipids; enhanced salt tolerance; improved membrane rigidity	[95]
*Nicotiana tabacum*	Overexpressing *AaSMO1*	400 mM NaCl for 0, 0.5, 1.5, 3, 6, 9, 24 and 72 h	Decreased the sensitivity of plants to dehydration stress; increased the content of total sterols	[96]
*Brassica oleracea, Brassica napus and Cakile maritima*		100 and 200 mM NaCl for 1 week	Increased the level of stigmasterol; enhanced the adaption of the membrane to salt stress	[97]
*Linum usitatissimum*	seeds were soaked with 200 ppm stigmasterol for 12 h	0, 100, 150 or 200 mM NaCl for 40 days	Decreased the drastic affect by NaCl; enhanced plant salinity tolerance	[98]
*Capsicum annuum*	150 ppm sitosterol	50, 100 or 200 mM NaCl	Offset the salinity stress damage; improved membrane stability and antioxidant enzyme activity	[99]
*Vitis vinifera*		UV-B (8.25 lW·cm^−2^, 16 h)	Increased the content of sitosterol and stigmasterol	[100]
*Vitis vinifera*		UV-B (33 lW·cm^−2^, 4 h)	Increased the content of triterpenoids 4.8-fold	[100]
*Olea europaea*		UV-B (6.5 kJ·m^−^^2^·day^−^^1^, 5 days)	No significant influence on the content of sterol	[101]
*Olea europaea*		UV-B (12.4 kJ·m^−^^2^·day^−^^1^, 5 days)	No significant influence on the content of sterol	[101]
*Withania somnifera*		UV-B (3.6 kJ·m^−2^·day^−1^)	Decreased the content of triterpenoids in leaf; increased triterpenoids levels in root	[102]
*Oryza sativa*	150 μM sitosterol for 20 days	UV-B (315 ± 20 nm, 6 h per day, 5 days)	Improved the growth of rice plants; enhanced tolerance of rice to UV-B stress	[103]
*Oryza sativa*		Water stress for 3, 6, 9 and 12 days	Up-regulated the level of stigmasterol, campesterol, β-sitosterol; decreased the activity of HMGR in rice	[104]
*Cucurbita pepo*		Five levels of drought stress on seed	Increased the content of plant sterols, especially β-sitosterol; inhibited the oil production of pumpkin seeds	[105]
*Oryza sativa*		Drought stress for 3, 6, 9 and 12 days	Increased the content of both free sterols and sterol ester; improved HMGR activity	[106]
*Camellia sinensis*		1, 8, and 15 days under water deprivation treatment	Increased sterols levels	[107]
*Cynodon dactylon*	*smt1* mutant	Drought stress for 7 days	Increased the content of cholesterol, putrescine (Put), spermidine (Spd), and spermine (Spm); improved drought tolerance	[68]
*Oryza sativa*	Knock-down of *OsSMT1* expression by RNA interference	Drought stress for 7 days	Increased the content of cholesterol, Put, Spd, and Spm; improved drought tolerance	[68]
*Trifolium repens*	120 μM sitosterol for 3 days	Drought stress for 7 days	Enhanced the drought tolerance and total antioxidant capacity.	[108]
*Triticum aestivum*	0, 25, 50, 100 mg·L^−1^ sitosterolapplied to wheat plants foliage	50% of crop evapotranspiration for 45 days	Offset the damage caused by drought to plants; improved yield.	[109]

**Table 2:** The regulation of the transcription factors to the plant sterols synthesis in plants.

Transcription Factor (TF)	TF Source	Plant	Regulation Type	Structural Gene	References
WsWRKY1	*Withania somnifera*	*Withania somnifera*	Positive	*HMGS, HMGR, SQS, SQE, CAS, C14-R, DWF1, 7DR-1, SSR1*, and *CYP710A1*	[126]
WsWRKY1	*Withania somnifera*	*Nicotiana tabacum*	Positive	*HMGR, FPPS, SQS SQE, CAS, SMT1*, and *CYP51*	[126]
WsWRKY1	*Withania somnifera*	*Solanum lycopersicum*	Positive	*SQS and SQE*	[127]
PqWRKY1	*Panax quinquefolius*	*Arabidopsis thaliana*	Positive	*HMGR, SQS1*, and *SQE2*	[128]
WsMYC2	*Withania somnifera*	*Tripterygium wilfordii*	Positive	*CYP710A* and *CAS*	[129]
TwMYC2a/TwMYC2b	*Tripterygium wilfordii*	*Tripterygium wilfordii*	Negative	*HMGR1*	[130]
ERF4	*Solanum lycopersicum*	*Solanum lycopersicum*	Positive	Nearly all genes of the cholesterol biosynthesis	[131]

## Data Availability

Not applicable.

## References

[1] Wollam J., Antebi A. (2011). Sterol regulation of metabolism, homeostasis, and development. Annu. Rev. Biochem..

[2] Hu Z., He B., Ma L., Sun Y., Niu Y., Zeng B. (2017). Recent Advances in Ergosterol Biosynthesis and Regulation Mechanisms in Saccharomyces cerevisiae. Indian J. Microbiol..

[3] Salehi B., Quispe C., Sharifi-Rad J., Cruz-Martins N., Nigam M., Mishra A.P., Konovalov D.A., Orobinskaya V., Abu-Reidah I.M., Zam W. (2020). Phytosterols: From Preclinical Evidence to Potential Clinical Applications. Front. Pharmacol..

[4] Ferrer A., Altabella T., Arró M., Boronat A. (2017). Emerging roles for conjugated sterols in plants. Prog. Lipid Res..

[5] Schaller H. (2003). The role of sterols in plant growth and development. Prog. Lipid Res..

[6] Simons K., Sampaio J.L. (2011). Membrane organization and lipid rafts. Cold Spring Harb Perspect. Biol..

[7] Rogowska A., Szakiel A. (2020). The role of sterols in plant response to abiotic stress. Phytochem. Rev..

[8] Bhat R.A., Panstruga R. (2005). Lipid rafts in plants. Planta.

[9] Lindsey K., Pullen M.L., Topping J.F. (2003). Importance of plant sterols in pattern formation and hormone signalling. Trends Plant Sci..

[10] Shimada T.L., Ueda T., Hara-Nishimura I. (2021). Excess sterol accumulation affects seed morphology and physiology in *Arabidopsis thaliana*. Plant Signal. Behav..

[11] Vriet C., Russinova E., Reuzeau C. (2013). From squalene to brassinolide: The steroid metabolic and signaling pathways across the plant kingdom. Mol. Plant.

[12] Ohyama K., Suzuki M., Kikuchi J., Saito K., Muranaka T. (2009). Dual biosynthetic pathways to phytosterol via cycloartenol and lanosterol in Arabidopsis. Proc. Natl. Acad. Sci. USA.

[13] De Vriese K., Pollier J., Goossens A., Beeckman T., Vanneste S. (2021). Dissecting cholesterol and phytosterol biosynthesis via mutants and inhibitors. J. Exp. Bot..

[14] Valitova J.N., Sulkarnayeva A.G., Minibayeva F.V. (2016). Plant Sterols: Diversity, Biosynthesis, and Physiological Functions. Biochemistry.

[15] Sonawane P.D., Pollier J., Panda S., Szymanski J., Massalha H., Yona M., Unger T., Malitsky S., Arendt P., Pauwels L. (2016). Plant cholesterol biosynthetic pathway overlaps with phytosterol metabolism. Nat. Plants.

[16] Atsumi G., Kagaya U., Tabayashi N., Matsumura T. (2018). Analysis of the mechanisms regulating the expression of isoprenoid biosynthesis genes in hydroponically grown *Nicotiana benthamiana* plants using virus-induced gene silencing. Sci. Rep..

[17] Babiychuk E., Navé P.B., Compagnon V., Suzuki M., Muranaka T., Van Montagu M., Kushnir S., Schaller H. (2008). Albinism and cell viability in cycloartenol synthase deficient Arabidopsis. Plant Sig. Behav..

[18] Wang X.T., Sun T.T., Sun J., Wang S., Zou L. (2021). Expression Analysis of Lanosterol synthase Gene in Dynamic Accumulation of Triterpenoids in Sanghuangporus baumii. Protein Pept. Lett..

[19] Nes W.D. (2011). Biosynthesis of cholesterol and other sterols. Chem. Rev..

[20] Schwenk E., Alexander G.J. (1958). Biogenesis of yeast sterols. II. Formation of ergosterol in yeast homogenates. Arch. Biochem. Biophys..

[21] Corey E.J., Matsuda S.P., Bartel B. (1993). Isolation of an Arabidopsis thaliana gene encoding cycloartenol synthase by functional expression in a yeast mutant lacking lanosterol synthase by the use of a chromatographic screen. Proc. Natl. Acad. Sci. USA.

[22] Kumar A., Fogelman E., Weissberg M., Tanami Z., Veilleux R.E., Ginzberg I. (2017). Lanosterol synthase-like is involved with differential accumulation of steroidal glycoalkaloids in potato. Planta.

[23] Gas-Pascual E., Berna A., Bach T.J., Schaller H. (2014). Plant oxidosqualene metabolism: Cycloartenol synthase-dependent sterol biosynthesis in Nicotiana benthamiana. PLoS ONE.

[24] Knoch E., Sugawara S., Mori T., Poulsen C., Fukushima A., Harholt J., Fujimoto Y., Umemoto N., Saito K. (2018). Third DWF1 paralog in Solanaceae, sterol Δ(24)-isomerase, branches withanolide biosynthesis from the general phytosterol pathway. Proc. Natl. Acad. Sci. USA.

[25] Tsukagoshi Y., Suzuki H., Seki H., Muranaka T., Ohyama K., Fujimoto Y. (2016). Ajuga Δ24-Sterol Reductase Catalyzes the Direct Reductive Conversion of 24-Methylenecholesterol to Campesterol. J. Biol. Chem..

[26] Zhang M., Wang C., Lin Q., Liu A., Wang T., Feng X., Liu J., Han H., Ma Y., Bonea D. (2015). A tetratricopeptide repeat domain-containing protein SSR1 located in mitochondria is involved in root development and auxin polar transport in Arabidopsis. Plant J..

[27] Choe S., Dilkes B.P., Gregory B.D., Ross A.S., Yuan H., Noguchi T., Fujioka S., Takatsuto S., Tanaka A., Yoshida S. (1999). The Arabidopsis *dwarf1* mutant is defective in the conversion of 24-methylenecholesterol to campesterol in brassinosteroid biosynthesis. Plant Physiol..

[28] Zheng X., Xiao Y., Tian Y., Yang S., Wang C. (2020). PcDWF1, a pear brassinosteroid biosynthetic gene homologous to AtDWARF1, affected the vegetative and reproductive growth of plants. BMC Plant Biol..

[29] Youn J.H., Kim T.W., Joo S.H., Son S.H., Roh J., Kim S., Kim T.W., Kim S.K. (2018). Function and molecular regulation of DWARF1 as a C-24 reductase in brassinosteroid biosynthesis in Arabidopsis. J. Exp. Bot..

[30] Sawai S., Ohyama K., Yasumoto S., Seki H., Sakuma T., Yamamoto T., Takebayashi Y., Kojima M., Sakakibara H., Aoki T. (2014). Sterol side chain reductase 2 is a key enzyme in the biosynthesis of cholesterol, the common precursor of toxic steroidal glycoalkaloids in potato. Plant Cell.

[31] Yasumoto S., Umemoto N., Lee H.J., Nakayasu M., Sawai S., Sakuma T., Yamamoto T., Mizutani M., Saito K., Muranaka T. (2019). Efficient genome engineering using Platinum TALEN in potato. Plant Biotechnol..

[32] Shi J., Gonzales R.A., Bhattacharyya M.K. (1996). Identification and characterization of an S-adenosyl-L-methionine: Delta 24-sterol-C-methyltransferase cDNA from soybean. J. Biol. Chem..

[33] Schaeffer A., Bronner R., Benveniste P., Schaller H. (2001). The ratio of campesterol to sitosterol that modulates growth in Arabidopsis is controlled by Sterol Methyltransferase 2;1. Plant J..

[34] Wang J., Liu J., Song Z., Nes W.D. (2008). Sterol C24-methyltransferase: Mechanistic studies of the C-methylation reaction with 24-fluorocycloartenol. Bioorg. Med. Chem. Lett..

[35] Pal S., Rastogi S., Nagegowda D.A., Gupta M.M., Shasany A.K., Chanotiya C.S. (2019). RNAi of Sterol Methyl Transferase1 Reveals its Direct Role in Diverting Intermediates Towards Withanolide/Phytosterol Biosynthesis in *Withania somnifera*. Plant Cell Physiol..

[36] Holmberg N., Harker M., Gibbard C.L., Wallace A.D., Clayton J.C., Rawlins S., Hellyer A., Safford R. (2002). Sterol C-24 methyltransferase type 1 controls the flux of carbon into sterol biosynthesis in tobacco seed. Plant Physiol..

[37] Nakamoto M., Schmit A.C., Heintz D., Schaller H., Ohta D. (2015). Diversification of sterol methyltransferase enzymes in plants and a role for β-sitosterol in oriented cell plate formation and polarized growth. Plant J..

[38] Carland F., Fujioka S., Nelson T. (2010). The sterol methyltransferases SMT1, SMT2, and SMT3 influence Arabidopsis development through nonbrassinosteroid products. Plant Physiol..

[39] De Storme N., De Schrijver J., Van Criekinge W., Wewer V., Dörmann P., Geelen D. (2013). GLUCAN SYNTHASE-LIKE8 and STEROL METHYLTRANSFERASE2 are required for ploidy consistency of the sexual reproduction system in Arabidopsis. Plant Cell.

[40] Neelakandan A.K., Nguyen H.T., Kumar R., Tran L.S., Guttikonda S.K., Quach T.N., Aldrich D.L., Nes W.D., Nguyen H.T. (2010). Molecular characterization and functional analysis of Glycine max sterol methyl transferase 2 genes involved in plant membrane sterol biosynthesis. Plant Mol. Biol..

[41] Bouvier F., Rahier A., Camara B. (2005). Biogenesis, molecular regulation and function of plant isoprenoids. Prog. Lipid Res..

[42] Rahier A. (2011). Dissecting the sterol C-4 demethylation process in higher plants. From structures and genes to catalytic mechanism. Steroids.

[43] Hu D., Gao Y.H., Yao X.S., Gao H. (2020). Recent advances in dissecting the demethylation reactions in natural product biosynthesis. Curr. Opin. Chem. Biol..

[44] Mialoundama A.S., Jadid N., Brunel J., Di Pascoli T., Heintz D., Erhardt M., Mutterer J., Bergdoll M., Ayoub D., Van Dorsselaer A. (2013). Arabidopsis ERG28 tethers the sterol C4-demethylation complex to prevent accumulation of a biosynthetic intermediate that interferes with polar auxin transport. Plant Cell.

[45] Ačimovič J., Rozman D. (2013). Steroidal triterpenes of cholesterol synthesis. Molecules.

[46] Morikawa T., Mizutani M., Aoki N., Watanabe B., Saga H., Saito S., Oikawa A., Suzuki H., Sakurai N., Shibata D. (2006). Cytochrome P450 CYP710A encodes the sterol C-22 desaturase in Arabidopsis and tomato. Plant Cell.

[47] Gutiérrez-García L., Arró M., Altabella T., Ferrer A., Boronat A. (2021). Structural and functional analysis of tomato sterol C22 desaturase. BMC Plant Biol..

[48] Morikawa T., Saga H., Hashizume H., Ohta D. (2009). CYP710A genes encoding sterol C22-desaturase in Physcomitrella patens as molecular evidence for the evolutionary conservation of a sterol biosynthetic pathway in plants. Planta.

[49] Laffaru Singpho N., Sharma J.G. (2021). Importance of Cytochrome P450 gene family from metabolite biosynthesis to stress tolerance: A review. IOP Conf. Ser. Earth Environ. Sci..

[50] Pandian B.A., Sathishraj R., Djanaguiraman M., Prasad P.V.V., Jugulam M. (2020). Role of Cytochrome P450 Enzymes in Plant Stress Response. Antioxidants.

[51] Mitu S.A., Ogbourne S.M., Klein A.H., Tran T.D., Reddell P.W., Cummins S.F. (2021). The P450 multigene family of Fontainea and insights into diterpenoid synthesis. BMC Plant Biol..

[52] Wei L.L., Chen W.C., Zhao W.C., Wang J., Wang B.R., Li F.J., Wei M.D., Guo J., Chen C.J., Zheng J.Q. (2020). Mutations and Overexpression of CYP51 Associated with DMI-Resistance in Colletotrichum gloeosporioides from Chili. Plant Dis..

[53] Zhang M., Song M., Cheng F., Yang Z., Davoudi M., Chen J., Lou Q. (2021). Identification of a putative candidate gene encoding 7-dehydrocholesterol reductase involved in brassinosteroids biosynthesis for compact plant architecture in Cucumber (*Cucumis sativus* L.). Theor. Appl. Genet..

[54] Han C., Yang P. (2015). Studies on the molecular mechanisms of seed germination. Proteomics.

[55] Yu L., Fan J., Zhou C., Xu C. (2021). Sterols are required for the coordinated assembly of lipid droplets in developing seeds. Nat. Commun..

[56] Wang H., Nagegowda D.A., Rawat R., Bouvier-Navé P., Guo D., Bach T.J., Chye M.L. (2012). Overexpression of Brassica juncea wild-type and mutant HMG-CoA synthase 1 in Arabidopsis up-regulates genes in sterol biosynthesis and enhances sterol production and stress tolerance. Plant Biotechnol. J..

[57] Baud S., Dichow N.R., Kelemen Z., d’Andréa S., To A., Berger N., Canonge M., Kronenberger J., Viterbo D., Dubreucq B. (2009). Regulation of HSD1 in seeds of *Arabidopsis thaliana*. Plant Cell Physiol..

[58] Moraes T.S., Dornelas M.C., Martinelli A.P. (2019). FT/TFL1: Calibrating Plant Architecture. Front. Plant Sci..

[59] Barthélémy D., Caraglio Y. (2007). Plant architecture: A dynamic, multilevel and comprehensive approach to plant form, structure and ontogeny. Ann. Bot..

[60] Wang Y., Li J. (2008). Molecular basis of plant architecture. Annu. Rev. Plant Biol..

[61] Wang M., Li P., Ma Y., Nie X., Grebe M., Men S. (2021). Membrane Sterol Composition in *Arabidopsis* thaliana Affects Root Elongation via Auxin Biosynthesis. Int. J. Mol. Sci..

[62] Schrick K., Debolt S., Bulone V. (2012). Deciphering the molecular functions of sterols in cellulose biosynthesis. Front. Plant Sci..

[63] Schrick K., Fujioka S., Takatsuto S., Stierhof Y.D., Stransky H., Yoshida S., Jürgens G. (2004). A link between sterol biosynthesis, the cell wall, and cellulose in Arabidopsis. Plant J..

[64] Rahim M.A., Jung H.J., Afrin K.S., Lee J.H., Nou I.S. (2018). Comparative transcriptome analysis provides insights into dwarfism in cherry tomato (*Solanum lycopersicum var. cerasiforme*). PLoS ONE.

[65] Klahre U., Noguchi T., Fujioka S., Takatsuto S., Yokota T., Nomura T., Yoshida S., Chua N.H. (1998). The Arabidopsis *DIMINUTO/DWARF1* gene encodes a protein involved in steroid synthesis. Plant Cell.

[66] Hong Z., Ueguchi-Tanaka M., Shimizu-Sato S., Inukai Y., Fujioka S., Shimada Y., Takatsuto S., Agetsuma M., Yoshida S., Watanabe Y. (2002). Loss-of-function of a rice brassinosteroid biosynthetic enzyme, C-6 oxidase, prevents the organized arrangement and polar elongation of cells in the leaves and stem. Plant J..

[67] Nomura T., Jager C.E., Kitasaka Y., Takeuchi K., Fukami M., Yoneyama K., Matsushita Y., Nyunoya H., Takatsuto S., Fujioka S. (2004). Brassinosteroid deficiency due to truncated steroid 5alpha-reductase causes dwarfism in the lk mutant of pea. Plant Physiol..

[68] Chen M., Chen J., Luo N., Qu R., Guo Z., Lu S. (2018). Cholesterol accumulation by suppression of *SMT1* leads to dwarfism and improved drought tolerance in herbaceous plants. Plant Cell Environ..

[69] Pereira A.M., Coimbra S. (2019). Advances in plant reproduction: From gametes to seeds. J. Exp. Bot..

[70] Suo X., Xu F., Tan K., Huang L., Bao C., Luo M. (2021). Functions of phytosterols in seed development of upland cotton (*Gossypium hirsutum* L.). Ind. Crops Prod..

[71] Liao P., Wang H., Wang M., Hsiao A.S., Bach T.J., Chye M.L. (2014). Transgenic tobacco overexpressing *Brassica juncea* HMG-CoA synthase 1 shows increased plant growth, pod size and seed yield. PLoS ONE.

[72] Jiao Z., Xu W., Zeng X., Xu X., Zhang M., Xia K. (2020). Obtusifoliol 14α-demethylase OsCYP51G1 is involved in phytosterol synthesis and affects pollen and seed development. Biochem. Biophys. Res. Commun..

[73] Han B., Yang N., Pu H., Wang T. (2018). Quantitative Proteomics and Cytology of Rice Pollen Sterol-Rich Membrane Domains Reveals Pre-established Cell Polarity Cues in Mature Pollen. J. Proteome Res..

[74] Moscatelli A., Idilli A.I. (2009). Pollen tube growth: A delicate equilibrium between secretory and endocytic pathways. J. Integr. Plant Biol..

[75] Liu P., Li R.L., Zhang L., Wang Q.L., Niehaus K., Baluska F., Samaj J., Lin J.X. (2009). Lipid microdomain polarization is required for NADPH oxidase-dependent ROS signaling in *Picea meyeri* pollen tube tip growth. Plant J..

[76] Moscatelli A., Gagliardi A., Maneta-Peyret L., Bini L., Stroppa N., Onelli E., Landi C., Scali M., Idilli A.I., Moreau P. (2015). Characterisation of detergent-insoluble membranes in pollen tubes of *Nicotiana tabacum* (L.). Biol. Open.

[77] Ozolina N.V., Gurina V.V., Nesterkina I.S., Nurminsky V.N. (2020). Variations in the content of tonoplast lipids under abiotic stress. Planta.

[78] Dufourc E.J. (2008). The role of phytosterols in plant adaptation to temperature. Plant Signal. Behav..

[79] Aboobucker S.I., Suza W.P. (2019). Why Do Plants Convert Sitosterol to Stigmasterol?. Front. Plant Sci..

[80] Renkova A.G., Valitova J., Schaller H., Minibayeva F.V. (2019). The homoeologous genes encoding C24-sterol methyltransferase 1 in *Triticum aestivum*: Structural characteristics and effects of cold stress. Biol. Plant..

[81] Wei S., Yang X., Huo G., Ge G., Liu H., Luo L., Hu J., Huang D., Long P. (2020). Distinct Metabolome Changes during Seed Germination of Lettuce (*Lactuca sativa* L.) in Response to Thermal Stress as Revealed by Untargeted Metabolomics Analysis. Int. J. Mol. Sci..

[82] Guo S., Klinkesorn U., Lorjaroenphon Y., Ge Y., Na Jom K. (2021). Effects of germinating temperature and time on metabolite profiles of sunflower (*Helianthus annuus* L.) seed. Food Sci. Nutr..

[83] Véron B., Billard C., Dauguet J.C., Hartmann M.A. (1996). Sterol composition of phaeodactylum tricornutum as influenced by growth temperature and light spectral quality. Lipids.

[84] Takahashi D., Imai H., Kawamura Y., Uemura M. (2016). Lipid profiles of detergent resistant fractions of the plasma membrane in oat and rye in association with cold acclimation and freezing tolerance. Cryobiology.

[85] Senthil-Kumar M., Wang K., Mysore K.S. (2013). AtCYP710A1 gene-mediated stigmasterol production plays a role in imparting temperature stress tolerance in *Arabidopsis thaliana*. Plant. Signal. Behav..

[86] Valitova J., Renkova A., Mukhitova F., Dmitrieva S., Beckett R.P., Minibayeva F.V. (2019). Membrane sterols and genes of sterol biosynthesis are involved in the response of Triticum aestivum seedlings to cold stress. Plant Physiol. Biochem..

[87] Gamel R., Elsayed A., Bashasha J., Haroun S.A. (2017). Priming Tomato Cultivars in β-sitosterol or Gibberellic Acid Improves Tolerance for Temperature Stress. Int. J. Bot..

[88] Rossi S., Huang B. (2022). Sitosterol-mediated Antioxidant Regulation to Enhance Heat Tolerance in Creeping Bentgrass. J. Am. Soc. Hortic. Sci..

[89] Surjus A., Durand M. (1996). Lipid changes in soybean root membranes in response to salt treatment. J. Exp. Bot..

[90] Blits K.C., Gallagher J.L. (1990). Effect of NaCl on lipid content of plasma membranes isolated from roots and cell suspension cultures of the dicot halophyte *Kosteletzkya virginica* (L.) Presl. Plant Cell Rep..

[91] López-Pérez L., Martínez-Ballesta Mdel C., Maurel C., Carvajal M. (2009). Changes in plasma membrane lipids, aquaporins and proton pump of broccoli roots, as an adaptation mechanism to salinity. Phytochemistry.

[92] Salama K., Mansour M., Ali F., Abou Hadid A. (2007). NaCl-induced changes in plasma membrane lipids and proteins of Zea mays L. cultivars differing in their response to salinity. Acta Physiol. Plant..

[93] Wu J., Seliskar D.M., Gallagher J.L. (2005). The response of plasma membrane lipid composition in callus of the halophyte *Spartina patens* (Poaceae) to salinity stress. Am. J. Bot..

[94] Salama K.H.A., Mansour M.M.F. (2015). Choline priming-induced plasma membrane lipid alterations contributed to improved wheat salt tolerance. Acta Physiol. Plant..

[95] Kerkeb L., Donaire J.P., Venema K., Rodríguez-Rosales M.P. (2001). Tolerance to NaCl induces changes in plasma membrane lipid composition, fluidity and H+-ATPase activity of tomato calli. Physiol. Plant.

[96] Singh A., Jindal S., Longchar B., Khan F., Gupta V. (2015). Overexpression of Artemisia annua sterol C-4 methyl oxidase gene, *AaSMO1*, enhances total sterols and improves tolerance to dehydration stress in tobacco. Plant Cell Tissue Organ Cult..

[97] Chalbi N., Martínez-Ballesta M.C., Youssef N.B., Carvajal M. (2015). Intrinsic stability of Brassicaceae plasma membrane in relation to changes in proteins and lipids as a response to salinity. J. Plant Physiol..

[98] Hashem H.A., Bassuony F., Hassanein R.A., Baraka D.M., Khalil R. (2011). Stigmasterol seed treatment alleviates the drastic effect of NaCL and improves quality and yield in flax plants. Aust. J. Crop Sci..

[99] Abu-Muriefah S.S. (2015). Effect Of Sitosterol On Growth, Metabolism Additionally, Protein Pattern Of Pepper (*Capsicum Annuum L*) Plants Grown Under Salt Stress Conditions. Intl. J. Agric. Crop Sci..

[100] Gil M., Pontin M., Berli F., Bottini R., Piccoli P. (2012). Metabolism of terpenes in the response of grape (*Vitis vinifera L.*) leaf tissues to UV-B radiation. Phytochemistry.

[101] Celeste Dias M., Pinto D., Correia C., Moutinho-Pereira J., Oliveira H., Freitas H., Silva A.M.S., Santos C. (2018). UV-B radiation modulates physiology and lipophilic metabolite profile in *Olea europaea*. J. Plant Physiol..

[102] Takshak S., Agrawal S.B. (2014). Secondary metabolites and phenylpropanoid pathway enzymes as influenced under supplemental ultraviolet-B radiation in *Withania somnifera Dunal*, an indigenous medicinal plant. J. Photochem. Photobiol. B..

[103] Shahzad R., Ewas M., Harlina P.W., Khan S.U., Zhenyuan P., Nie X., Nishawy E. (2021). β-Sitosterol differentially regulates key metabolites for growth improvement and stress tolerance in rice plants during prolonged UV-B stress. J. Genet. Eng. Biotechnol..

[104] Kumar M.S., Ali K., Dahuja A., Tyagi A. (2015). Role of phytosterols in drought stress tolerance in rice. Plant Physiol. Biochem..

[105] Zeynali M., Maleki Zanjani B., Moradi P., Shekari F., Niazkhani S.M. (2020). Effects of drought stress on phytosterols content and expression of key genes involved in Beta sitosterol biosysthesis pathway in medicinal plant pumpkin (*Cucurbita pepo* L.). J Iran. J. Med. Aromat. Plants Res..

[106] Kumar M.S.S., Mawlong I., Ali K., Tyagi A. (2018). Regulation of phytosterol biosynthetic pathway during drought stress in rice. Plant Physiol. Biochem..

[107] Chen M., Zhu X., Zhang Y., Du Z., Chen X., Kong X., Sun W., Chen C. (2020). Drought stress modify cuticle of tender tea leaf and mature leaf for transpiration barrier enhancement through common and distinct modes. Sci. Rep..

[108] Li Z., Cheng B., Yong B., Liu T., Peng Y., Zhang X., Ma X., Huang L., Liu W., Nie G. (2019). Metabolomics and physiological analyses reveal β-sitosterol as an important plant growth regulator inducing tolerance to water stress in white clover. Planta.

[109] Elkeilsh A., Awad Y.M., Soliman M.H., Abu-Elsaoud A., Abdelhamid M.T., El-Metwally I.M. (2019). Exogenous application of β-sitosterol mediated growth and yield improvement in water-stressed wheat (Triticum aestivum) involves up-regulated antioxidant system. J. Plant Res..

[110] Baker N.R., Long S.P., Ort D.R. (1988). Photosynthesis and temperature, with particular reference to effects on quantum yield. Symp. Soc. Exp. Biol..

[111] Airaki M., Leterrier M., Mateos R.M., Valderrama R., Chaki M., Barroso J.B., Del Río L.A., Palma J.M., Corpas F.J. (2012). Metabolism of reactive oxygen species and reactive nitrogen species in pepper (*Capsicum annuum* L.) plants under low temperature stress. Plant Cell Environ..

[112] Mathur M. (2012). Variations in phytosterol composition in Corchorus depressus and their relation with bottom-up, top-down and plant metabolites. J. Nat. Prod..

[113] Hennessey T.M. (1992). Effects of membrane plant sterols on excitable cell functions. Comp. Biochem. Physiol. C Comp. Pharmacol. Toxicol..

[114] Grosjean K., Mongrand S., Beney L., Simon-Plas F., Gerbeau-Pissot P. (2015). Differential effect of plant lipids on membrane organization: Specificities of phytosphingolipids and phytosterols. J. Biol. Chem..

[115] Munns R., Tester M. (2008). Mechanisms of salinity tolerance. Annu. Rev. Plant Biol..

[116] El Sabagh A., Islam M.S., Skalicky M., Ali Raza M., Singh K., Anwar Hossain M., Hossain A., Mahboob W., Iqbal M.A., Ratnasekera D. (2021). Salinity Stress in Wheat (*Triticum aestivum* L.) in the Changing Climate: Adaptation and Management Strategies. Front. Agron..

[117] Kakar N., Jumaa S.H., Redoña E.D., Warburton M.L., Reddy K.R. (2019). Evaluating rice for salinity using pot-culture provides a systematic tolerance assessment at the seedling stage. Rice.

[118] Guo Q., Liu L., Barkla B.J. (2019). Membrane Lipid Remodeling in Response to Salinity. Int. J. Mol. Sci..

[119] Ries G., Heller W., Puchta H., Sandermann H., Seidlitz H.K., Hohn B. (2000). Elevated UV-B radiation reduces genome stability in plants. Nature.

[120] Teramura A.H., Sullivan J.H. (1994). Effects of UV-B radiation on photosynthesis and growth of terrestrial plants. Photosynth. Res..

[121] Inostroza-Blancheteau C., Reyes-Díaz M., Arellano A., Latsague M., Acevedo P., Loyola R., Arce-Johnson P., Alberdi M. (2014). Effects of UV-B radiation on anatomical characteristics, phenolic compounds and gene expression of the phenylpropanoid pathway in highbush blueberry leaves. Plant Physiol. Biochem..

[122] Warner R., Wu B.S., MacPherson S., Lefsrud M. (2021). A Review of Strawberry Photobiology and Fruit Flavonoids in Controlled Environments. Front. Plant Sci..

[123] Berli F.J., Bottini R. (2013). UV-B and abscisic acid effects on grape berry maturation and quality. J. Berry Res..

[124] Matus J.T. (2016). Transcriptomic and Metabolomic Networks in the Grape Berry Illustrate That it Takes More Than Flavonoids to Fight Against Ultraviolet Radiation. Front. Plant Sci..

[125] Nezhadahmadi A., Prodhan Z.H., Faruq G. (2013). Drought tolerance in wheat. Sci. World J..

[126] Dhar N., Rana S., Razdan S., Bhat W.W., Hussain A., Dhar R.S., Vaishnavi S., Hamid A., Vishwakarma R., Lattoo S.K. (2014). Cloning and functional characterization of three branch point oxidosqualene cyclases from *Withania somnifera* (L.) dunal. J. Biol. Chem..

[127] Singh A.K., Kumar S.R., Dwivedi V., Rai A., Pal S., Shasany A.K., Nagegowda D.A. (2017). A WRKY transcription factor from *Withania somnifera* regulates triterpenoid withanolide accumulation and biotic stress tolerance through modulation of phytosterol and defense pathways. New Phytol..

[128] Sun Y., Niu Y., Xu J., Li Y., Luo H., Zhu Y., Liu M., Wu Q., Song J., Sun C. (2013). Discovery of WRKY transcription factors through transcriptome analysis and characterization of a novel methyl jasmonate-inducible PqWRKY1 gene from *Panax quinquefolius*. Plant Cell Tissue Organ Cul..

[129] Sharma A., Rather G.A., Misra P., Dhar M.K., Lattoo S.K. (2019). Jasmonate responsive transcription factor WsMYC2 regulates the biosynthesis of triterpenoid withanolides and phytosterol via key pathway genes in Withania somnifera (L.) Dunal. Plant Mol. Biol..

[130] Huo Y., Zhang J., Zhang B., Chen L., Zhang X., Zhu C. (2021). MYC2 Transcription Factors TwMYC2a and TwMYC2b Negatively Regulate Triptolide Biosynthesis in *Tripterygium wilfordii* Hairy Roots. Plants.

[131] Thagun C., Imanishi S., Kudo T., Nakabayashi R., Ohyama K., Mori T., Kawamoto K., Nakamura Y., Katayama M., Nonaka S. (2016). Jasmonate-Responsive ERF Transcription Factors Regulate Steroidal Glycoalkaloid Biosynthesis in Tomato. Plant Cell Physiol..

[132] Tian Y., Lu X.Y., Peng L.S., Fang J. (2006). The structure and function of plant WRKY transcription factors. Yi Chuan = Hereditas.

[133] Rushton P.J., Somssich I.E., Ringler P., Shen Q.J. (2010). WRKY transcription factors. Trends. Plant Sci..

[134] Wani S.H., Anand S., Singh B., Bohra A., Joshi R. (2021). WRKY transcription factors and plant defense responses: Latest discoveries and future prospects. Plant Cell Rep..

[135] Yanfang Y., Kaikai Z., Liying Y., Xing L., Ying W., Hongwei L., Qiang L., Duanfen C., Deyou Q. (2018). Identification and characterization of MYC transcription factors in *Taxus* sp.. Gene.

[136] Kazan K., Manners J.M. (2013). MYC2: The master in action. Mol. Plant.

[137] Peñuelas M., Monte I., Schweizer F., Vallat A., Reymond P., García-Casado G., Franco-Zorrilla J.M., Solano R. (2019). Jasmonate-Related MYC Transcription Factors Are Functionally Conserved in Marchantia polymorpha. Plant Cell.

[138] Licausi F., Ohme-Takagi M., Perata P. (2013). APETALA2/Ethylene Responsive Factor (AP2/ERF) transcription factors: Mediators of stress responses and developmental programs. New Phytol..

[139] Debbarma J., Sarki Y.N., Saikia B., Boruah H.P.D., Singha D.L., Chikkaputtaiah C. (2019). Ethylene Response Factor (ERF) Family Proteins in Abiotic Stresses and CRISPR-Cas9 Genome Editing of ERFs for Multiple Abiotic Stress Tolerance in Crop Plants: A Review. Mol. Biotechnol..

[140] Xie Z., Nolan T.M., Jiang H., Yin Y. (2019). AP2/ERF Transcription Factor Regulatory Networks in Hormone and Abiotic Stress Responses in Arabidopsis. Front Plant Sci..

[141] Sears M.T., Zhang H., Rushton P.J., Wu M., Han S., Spano A.J., Timko M.P. (2014). NtERF32: A non-NIC2 locus AP2/ERF transcription factor required in jasmonate-inducible nicotine biosynthesis in tobacco. Plant Mol. Biol..

[142] De Boer K., Tilleman S., Pauwels L., Vanden Bossche R., De Sutter V., Vanderhaeghen R., Hilson P., Hamill J.D., Goossens A. (2011). APETALA2/ETHYLENE RESPONSE FACTOR and basic helix-loop-helix tobacco transcription factors cooperatively mediate jasmonate-elicited nicotine biosynthesis. Plant J..

[143] Xu L., Simoni R.D. (2003). The inhibition of degradation of 3-hydroxy-3-methylglutaryl coenzyme A (HMG-CoA) reductase by sterol regulatory element binding protein cleavage-activating protein requires four phenylalanine residues in span 6 of HMG-CoA reductase transmembrane domain. Arch. Biochem. Biophys..

[144] Gnad F., Gunawardena J., Mann M. (2011). PHOSIDA 2011: The posttranslational modification database. Nucleic Acids Res..

[145] Heazlewood J.L., Durek P., Hummel J., Selbig J., Weckwerth W., Walther D., Schulze W.X. (2008). PhosPhAt: A database of phosphorylation sites in Arabidopsis thaliana and a plant-specific phosphorylation site predictor. Nucleic Acids res..

[146] Erffelinck M.L., Goossens A. (2018). Review: Endoplasmic Reticulum-Associated Degradation (ERAD)-Dependent Control of (Tri)terpenoid Metabolism in Plants. Planta Med..

[147] Shimada T.L., Shimada T., Okazaki Y., Higashi Y., Saito K., Kuwata K., Oyama K., Kato M., Ueda H., Nakano A. (2019). HIGH STEROL ESTER 1 is a key factor in plant sterol homeostasis. Nat. Plants.

[148] Bouvier-Navé P., Berna A., Noiriel A., Compagnon V., Carlsson A.S., Banas A., Stymne S., Schaller H. (2010). Involvement of the phospholipid sterol acyltransferase1 in plant sterol homeostasis and leaf senescence. Plant Physiol..

[149] Carland F.M., Fujioka S., Takatsuto S., Yoshida S., Nelson T. (2002). The identification of CVP1 reveals a role for sterols in vascular patterning. Plant Cell.

[150] Peng L., Kawagoe Y., Hogan P., Delmer D. (2002). Sitosterol-beta-glucoside as primer for cellulose synthesis in plants. Science.

[151] Berger A., Ralet M.C., Akary E., Sallé C., Grandjean O., Debeaujon I., North H.M. (2021). Sterol Glucosyltransferases Tailor Polysaccharide Accumulation in Arabidopsis Seed Coat Epidermal Cells. Cells.

